# Extending QGroundControl for Automated Mission Planning of UAVs

**DOI:** 10.3390/s18072339

**Published:** 2018-07-18

**Authors:** Cristian Ramirez-Atencia, David Camacho

**Affiliations:** Department of Computer Science, Universidad Autónoma de Madrid, 28049 Madrid, Spain; david.camacho@uam.es

**Keywords:** decision support system, ground control station, mission planning, multi-objective optimization, QGroundControl, unmanned aerial vehicles

## Abstract

Unmanned Aerial Vehicle (UAVs) have become very popular in the last decade due to some advantages such as strong terrain adaptation, low cost, zero casualties, and so on. One of the most interesting advances in this field is the automation of mission planning (task allocation) and real-time replanning, which are highly useful to increase the autonomy of the vehicle and reduce the operator workload. These automated mission planning and replanning systems require a Human Computer Interface (HCI) that facilitates the visualization and selection of plans that will be executed by the vehicles. In addition, most missions should be assessed before their real-life execution. This paper extends QGroundControl, an open-source simulation environment for flight control of multiple vehicles, by adding a mission designer that permits the operator to build complex missions with tasks and other scenario items; an interface for automated mission planning and replanning, which works as a test bed for different algorithms, and a Decision Support System (DSS) that helps the operator in the selection of the plan. In this work, a complete guide of these systems and some practical use cases are provided.

## 1. Introduction

The use of Unmanned Aerial Vehicles (UAVs), also referred to as drones, has highly increased in the last decade, becoming very popular in many applications including traffic monitoring [1], agriculture [2] or disaster and crisis management [3], since they avoid risking human lives while their manageability permits reaching areas that are hard to access. These vehicles are usually controlled by a number of operators inside one or more Ground Control Station (GCSs), depending on the size of the mission.

Automated mission planning over a swarm of UAVs remains to date a challenging research trend in regards to this particular type of aircraft. This problem involves generating tactical goals, commanding vehicles, risk avoidance, coordination and timing. Currently, UAVs are controlled remotely by human operators using rudimentary planning systems, such as pre-configured plans, classical planners that are not able to cope with the entire complexity of the problem or manually provided schedules. Some recent works [4,5] have provided more efficient approaches to solve the Multi-UAV Cooperative Mission Planning Problem (MCMPP) considering several features of the problem such as time constraints, fuel constraints, sensor constraints, etc. Due to its complexity and multiple conflicting criteria (e.g., makespan, cost or risk of the mission), multi-objective solvers such as Multi-Objective Evolutionary Algorithm (MOEAs) have been used in these works.

One of the most challenging problems in this field is mission replanning, which implies a new planning for the previous mission plan due to certain incidences, such as a vehicle or sensor failure or a new task arrival, during the real-time execution of the mission. A few recent works have developed systems that deal with automated mission replanning, based on a repair of the previous plan [6], or performing a full replanning of the mission in a limited runtime [7].

Due to the complexity and multiple conflicting objectives of this problem, several non-dominated solutions (i.e., the Pareto Optimal Frontier (POF)) are obtained. This situation hinders the process of decision making for the operator when selecting the final plan. In order to reduce his/her workload, a Decision Support System (DSS) can be provided to help the operator in the plan selection. This DSS can work in two steps: first, inside the mission planning algorithm, focusing the search of solutions on the most relevant ones, which can be made using a knee point-based MOEA [8]. Secondly, once the most relevant solutions are returned, the solutions are ranked using some Multi-criteria Decision Making (MCDM) technique based on the operator preferences and filtered based on the similarity of the obtained solutions.

All of these techniques developed to solve the mission planning and replanning problems must be properly tested by expert operators in a simulated environment before they are considered apt for real UAV missions. In this work, QGroundControl [9], an open-source ground control station simulator, has been extended by adding an automated planning interface, so this framework can work as a test bed for mission planning and replanning algorithms, and also for decision making methods. This extension allows operators to automatically plan a mission, simulate this plan and then perform a replanning during the execution.

**Figure 1:**
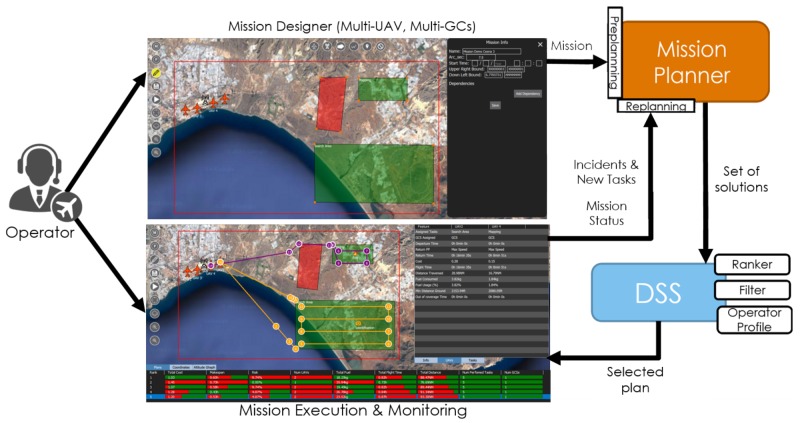
Architecture of the framework extended from QGroundControl, including mission (re)planning and decision support.

Additionally, in order to ease the entry of the definition of the mission and the scenario, a graphical mission designer has been built inside QGroundControl. This designer permits creating a mission with all its elements (UAVs, tasks, GCSs, No Flight Zones (NFZs), etc.). After that, the mission can be automatically planned and the generated plans can be visualized. Finally, one of these plans can be executed, and the UAVs are monitored all together. The architecture of the proposed framework is presented in Figure 1. This extended tool is not publicly accessible at the moment due to confidential issues (if the reader is interested in testing the current version of the framework, please contact the corresponding author).

The following sections are as follows: Section 2 provides a background and related works on ground control station frameworks and test bed interfaces. Section 3 describes the mission designer and its components. Section 4 explains the mission planning problem and how the automated mission planner and the DSS have been integrated within QGroundControl. Section 5 explains the mission replanning problem and how it has been integrated in the simulation environment. Section 6 presents some use cases that have been performed to prove the functionalities developed by creating, planning, simulating and replanning a mission. Finally, Section 7 draws some conclusions and future work.

## 2. Related Work

In the last few years, some works have proposed UAV simulation environments for supervisory flight control [10], coordination [11] or training [12].

When working with simulation and control of UAVs, there are two types of software that must be differentiated: the autopilot software and the GCS software. The autopilot automatically controls the trajectory of the UAV and can provide the telemetry of the vehicle. The most known autopilot simulators are ArduPilot [13] and PX4 [14].

On the other hand, GCS software focuses on the operator side, providing flight control and manual path planning of one or multiple vehicles. In order to communicate these GCSs to the autopilots, a communication protocol is required. The most used protocol, able to provide communication with both ArduPilot and PX4, is MAVLink [15]. This protocol is used in the most known GCS tools, such as MAVProxy [16], Mission Planner [17], APM Planner 2 [18], UgCS [19] and QGroundControl [9]. QGroundControl is the only one of them that permits the control of multiple UAVs simultaneously, although UgCS provides a much more proficient interface with many features such as NFZs and immersive 3D simulation.

One of the most popular open-source software for small drones is Paparazzi UAV [20], which provides both the autopilot and the GCS tools. It is a very complete framework and also allows control of multiple vehicles simultaneously.

QGroundControl is open source and provides full ground station support and flight control and configuration for multiple UAVs through MAVLink communications, allowing one to control both ArduPilot and PX4 vehicles. The main power of QGroundControl is that it provides easy and straightforward usage for beginners, while still delivering high-end feature support for experienced users. It has an easy-to-use path planning interface (through waypoint insertion) for autonomous flight. It also allows flight map display showing vehicle position, flight track, waypoints, vehicle instruments and video streaming.

Nevertheless, QGroundControl, as well as the rest of the GCS software, only permits one to create manual plans by waypoint insertion for each UAV, i.e., no automated planning algorithm is provided within the framework. It does not allow one to create tasks, nor NFZs in order to create a mission, which could be automatically planned. On the other hand, QGroundControl only permits one to see the waypoint plan of one UAV at a time, so for multi-UAV missions, it is complicated to monitor all vehicles at once.

A test bed interface (which has been used in many works especially for providing different artificial intelligence algorithms for games [21] and also for flight control [22]) for automated mission planning and replanning is a novel requirement that has not been so far implemented inside GCSs. This interface must allow, through a communications protocol, the use of different automated mission planning and replanning algorithms. This paper provides an extension of QGroundControl, providing this test bed and all the lacking capabilities mentioned.

## 3. Mission Designer

A mission is composed of a set of objectives to be performed by a swarm of UAVs. These objectives are composed by one or more tasks, where a task is indivisible and must be performed within a specific time interval using some sensors carried by the vehicle. These tasks may have some dependencies between them, restricting the order of the tasks. Each mission should be performed in a specific geographic zone or scenario, where there could be some NFZs that must be avoided by the vehicles. In addition, one or more GCSs control the swarm of UAVs.

**Figure 2:**
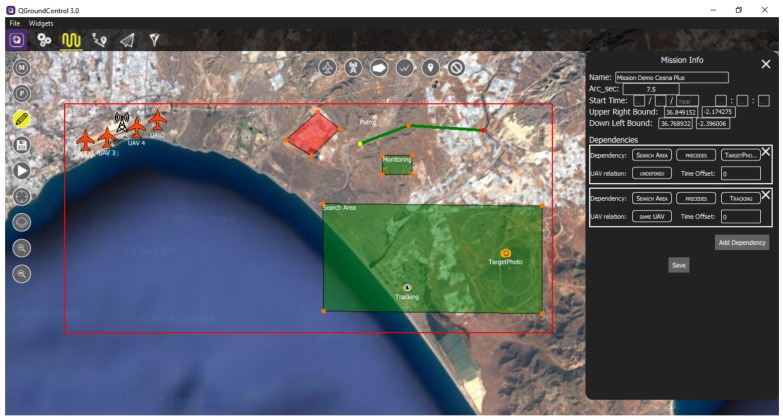
Mission designer in QGroundControl for adding new elements.

In order to permit the operators to create new missions and fulfill them with all the elements involved in the mission (tasks, vehicles, GCSs, etc.), a mission designer has been developed (see Figure 2). The integration of these new functionalities into QGroundControl requires the modification of this software in order to extend its functionality. For this, a new tab has been added in the main menu of the QGroundControl (represented in Figure 2 with a looped path in the top bar). This tab is used both for the mission designer and the rest of functionalities added in the following sections.

The mission designer permits one to create new missions or read already created ones and provides a set of tools for adding different elements to the mission scenario. In the following subsections, we explain how to create a new mission and how to add the different elements to it using the mission designer (represented with a pencil in Figure 2).

### 3.1. Creating New Missions

To create a new mission, there are several parameters of the mission that must be provided by the operator:

- The name of the mission: This name will be used later to identify this mission when reading it.
- The bounds of the mission scenario: The upper-right and bottom-left bounds that identify the limits of the mission, given in latitude and longitude degrees.
- The arc-seconds of the elevation map to be used by the mission planner. The possible values are 30, 15 and 7.5 arc-seconds.
- The start time of the mission (optional), expressed as date and time. If not provided, the time will be taken from the CPU clock when the simulation starts.

After fulfilling these values, the mission will be created, and an empty scenario inside the defined bounds will be presented.

### 3.2. Adding a New UAV

To add a UAV, the first button in the top of the scenario, represented as a plane in Figure 2, is used. When adding a new vehicle, some properties must be considered:

- The name of the UAV.
- The initial amount of fuel of the UAV, expressed in kg.
- The position of the UAV (latitude, longitude and altitude in ft).
- The (optional) end position for the departure runway of the UAV, where it must go when taking off.
- The (optional) start position for the landing track of the UAV, where it must first go when landing.
- The (optional) end position of the UAV, where it must land when the mission ends.
- The (optional) start and end times of use of the UAV. These are expressed as a date and time and can only be used when the start time of the mission has been defined.
- The type of the UAV. This property defines the type of vehicle used (e.g., HALE, MALE, URAV, UCAV, etc.). This property is comprised of a set of characteristics of the UAV:
  – The mass of the vehicle (in kg).
  – The maximum fuel capacity (in kg).
  – The cost per hour.
  – The maximum altitude limit (in ft).
  – The maximum speed limit (in knots).
  – The maximum flight time (in hours).
  – The maximum range or distance (in NM).
  A set of flight profiles, defining the performance of the vehicle in terms of speed (in knots), fuel consumption (in kg/h) and altitude (in ft) or angle of climb/descent (in degrees). In this work, the flight profiles considered for every vehicle are a minimum consumption profile, a maximum speed profile, a climb profile and a descent profile.
- The configuration of the UAV. This property defines the configuration of the vehicle, specifically the set of sensors carried by it.

### 3.3. Adding a New GCS

This function is represented by the second button in the top of the scenario in Figure 2. When adding a new GCS, some properties must be fulfilled:

- The name of the GCS.
- The position of the GCS (latitude, longitude and altitude in ft).
- The type of GCS. This property defines the type of station used, which is comprised of a set of characteristics:
  – The range of communications (in NM).
  – The maximum number of vehicles that the station can control simultaneously.
  – The type of vehicles that the station can control.

**Figure 3:**
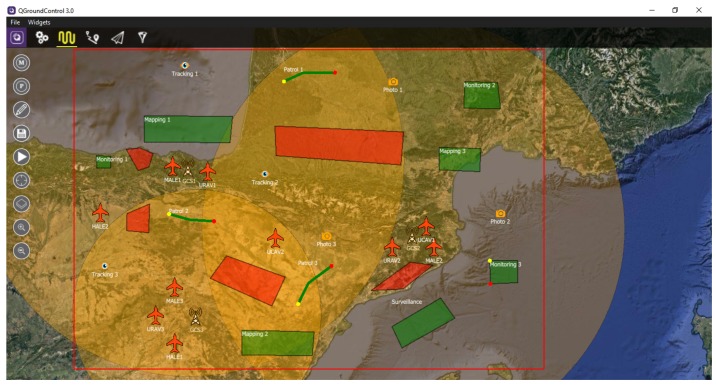
Range of Ground Control Stations (GCSs) represented as translucent orange circles.

To facilitate the creation of the mission, GCSs can show a translucent orange circle centered on the station and with a radius within its range, graphically representing the range of the GCS. This is shown in Figure 3.

### 3.4. Adding an Objective or Task

Adding objectives is performed through the third, fourth and fifth buttons at the top of the scenario, depending on whether the location of the objective is a polygon zone, a path or a point, respectively.

When adding a polygon objective, the operator will mark in order the vertices of the zone. This works similarly for the path objective, where the operator marks in order the points of the path. Point objectives, on the other hand, just need to select the single location on the map. Depending on the type of objective, a different figure will be used to represent the point objective (a fire flame for fire extinguishing, a camera for target photographing or an eye for target acquisition).

Objectives have different properties, including:

- The name of the objective.
- The (optional) start and end times of the objective. These are expressed as a date and time and can only be used when the start time of the mission has been defined.
- The (optional) duration of the objective. This can only be used with zone and point objectives. When provided, loitering around the zone or the point will be performed during the specified duration.
- Whether the objective is mandatory or not.
- Whether Line of Sight (LOS) must be maintained during the objective performance or not.
- The (optional) entry and exit points of the zone objective (expressed in latitude and longitude). These can only be used in zone objectives.
- The vertices of the zone or the path (expressed in latitude and longitude).
- The position of the point (expressed in latitude and longitude). Whether this or the previous property is used, but not both.
- The type of objective. This property defines the type of objectives (e.g., target photographing, escorting an individual, fire extinguishing, etc.). Depending on this type, an objective may comprise one or more tasks, each one needing a specific sensor for its performance. Depending on the type of task, it must be performed by just one vehicle (e.g., tracking or photographing) or could be performed by several (e.g., mapping or surveillance). In addition, some time or vehicle dependency may be established between the tasks of the objective. These dependencies are discussed in Section 3.6.

### 3.5. Adding an NFZ

An NFZ can be added using the sixth button at the top of the scenario. When adding a new NFZ, the operator will mark in order the vertices of the zone. This element and the rest of the elements explained in previous sections can be dragged and, thus, their positions and vertices updated. In addition, each vertex can be also dragged. On the other hand, any element of the mission scenario can be deleted by just right clicking over it.

### 3.6. Adding Objective Dependencies

When first entering edit mode, a general Mission Info panel appears on the right, where the initial definition of the mission (bounds, start time, etc.) can be modified. In this panel, dependencies between objectives can also be added with the Add Dependency button.

A dependency will consist of the following elements:

- First objective involved in the dependency.
- The type of dependency. The different types are defined in Allen interval algebra (see Table 1).
- Second objective involved in the dependency.
- UAV relation between objectives. This value may be undefined or provided if both objectives must be performed by the same UAV or by different UAVs.
- Time offset. This property defines the time offset applied between both objectives when considering the dependency (e.g., if an objective precedes another one, then the time offset sets the minimum duration that must pass between the end of the first objective and the start of the second).

## 4. Automated Mission Planner and DSS

The MCMPP consists of assigning each task of the mission to the vehicle(s) performing it and the order of performance and to each vehicle the GCS controlling it. In addition, it is also necessary to specify the flight profiles used by the UAVs in each path, as well as the sensor used in each task, as there could be several sensors on the UAV able to perform the task.

On the other hand, there exists a set of constraints that must be fulfilled to assure the validity of the solutions. These constraints include temporal constraints implying the start and end times of tasks, path constraints assuring that vehicles avoid NFZs in their paths, coverage constraints assuring that the UAV is inside the range of the GCS controlling it, LOS is maintained, etc. More information about this problem is presented in [5].

In addition, the MCMPP is a Multi-Objective Optimization Problem (MOP), as there are multiple variables that must be optimized, including the makespan or end time of the mission, the cost of the mission, the risk, the total fuel consumption of vehicles in the mission, the distance traversed, the flight time, the number of UAVs used and the number of tasks performed. When solving this problem, most of the existing algorithms focus on the approximation of the POF. Nevertheless, when the entire POF comprises a large number of solutions, the process of decision making to select one appropriate solution becomes a difficult task for the Decision Maker (DM). Sometimes, the DM provides a priori information about his/her preferences, which can be used in the optimization process. However, very often, the DM does not provide this information, and it is necessary to consider other approaches for filtering the number of solutions.

Moreover, a DSS is necessary to help the operator in the process of selection of the final plan. This system should provide at least a ranking system and a filtering system. The ranking system considers the operator profile, which provides for each decision variable an intensity factor: very low, low, medium, high or very high. Different MCDM methods have been developed over the years for this purpose [23].

Once the solutions are ranked, a filtering system based on the distance between the solutions (i.e., the variables of the encoding: the assignments, orders, etc.) is used to erase similar solutions. This distance function must consider the importance of each variable, where assignments are the most important variable, while flight profiles are the least important.

**Figure 4:**
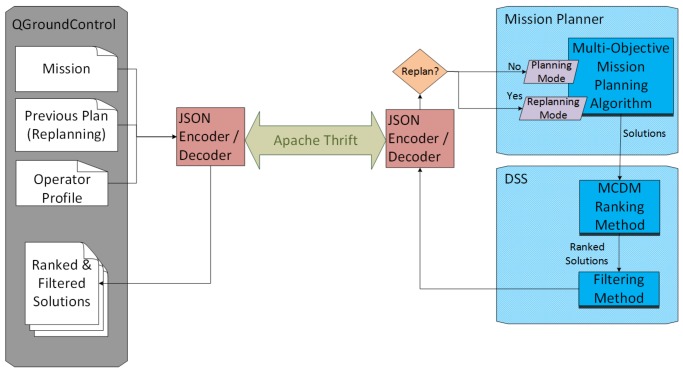
Architecture of the test bed interface for mission planning and decision support.

For all this process, since the mission is provided until the ranked and filtered solutions are returned, a test bed interface has been designed. The communication interface between QGroundControl and the planning and decision algorithms has been implemented using Apache Thrift (https://thrift.apache.org/). This frameworks permits an easy communication with most known programming languages. The interface sends the mission and the operator profile extracted from QGroundControl as a JSON message. This message includes the different parameters of each element of the mission explained in Section 3. On the other hand, the message returned by the algorithm must contain a ranked list of solutions, where each solution defines the assignments for each task, including the flight profile and sensors used; the GCS assignment, final path and performance variables (fuel consumption, flight time, etc.) for each UAV and the values of the optimization objectives and risk factors of the problem. The architecture of these modules is represented in Figure 4.

### 4.1. Operator Profile

Every operator using the QGroundControl has a profile defining the different constraints, fitness and ranking variables predefined. This profile includes the following settings:

- Whether all tasks must be performed or not.
- Minimum and risk factor distance from the ground: These variables define the interval for the risk factor distance from the ground, where the minimum represents a risk of 100% and values higher than the risk represent a 0% risk.
- Maximum and risk factor percentage of fuel usage: These variables define the interval for the risk factor of fuel usage per UAV, where the maximum represents a risk of 100% and values lower than the risk represent a 0% risk.
- Minimum and risk factor of distance between vehicles: These variables define the interval for the risk factor distance between UAVs, where the minimum represents a risk of 100% and values higher than the risk represent a 0% risk.
- Minimum and maximum risky factor of time out of GCS coverage: These variables define the interval for the risk factor of time out of GCS coverage, where the minimum represents a risk of 0% and the maximum risk represents a 100% risk.

On the other hand, the operator must also define the importance (very low, low, medium, high or very high) of the ranking variables to be used by the DSS. These variables include:

- Makespan or end time of the mission.
- Total cost of the mission.
- Total fuel consumption of vehicles in the mission.
- Total flight time of the vehicles in the mission.
- Total distance traversed by the vehicles in the mission.
- Risk of high fuel usage. This considers the UAVs that finish the mission with low fuel.
- Risk of low distance from the ground. This considers the vehicles that fly near the ground (depending on the route and the altitude of the adopted flight profile).
- Risk of GCS coverage loss. This considers UAVs that fly out of the coverage or LOS of the GCSs controlling them.
- Risk of UAV closeness. This considers vehicles that fly close to each other, which intuitively depends on the time constraints between concurrently-performed tasks and eventual spatial overlaps among routes/flight profiles.
- Number of UAVs employed in the mission.
- Number of tasks performed. This is considered when some tasks or objectives are not mandatory.
- Number of GCSs employed in the mission.

### 4.2. Mission Planning

Once a mission is defined, the mission planner (button “P” on the left panel in Figure 2) can be executed in order to find plans for this mission. Additionally, the operator preferences defined in Section 4.1 can be adapted for this concrete mission. Apart from the preferences explained before, the operator can also specify some constraints for the mission:

- Maximum makespan: The maximum valid makespan. Plans with higher makespan will be rejected from the solutions.
- Maximum cost: The maximum valid cost. Plans with higher cost will be rejected from the solutions.
- Maximum flight time: The maximum valid flight time. Plans with higher flight time will be rejected from the solutions.
- Maximum fuel consumption: The maximum valid fuel consumption. Plans with higher fuel consumption will be rejected from the solutions.
- Maximum distance traversed: The maximum valid distance traversed. Plans with a higher distance traversed will be rejected from the solutions.

Once this information is completed, it is encoded as a JSON message and sent through the Thrift interface to the automated mission planner, which must detect it as pre-planning and deal with multiple objectives.

### 4.3. DSS Ranking and Filtering

After the mission planner finishes, the DSS will take the solutions obtained and rank them according to the ranking criteria defined by the operator using some MCDM method. Then, the DSS will filter the solutions that are very similar (only differ in the flight profile used in some path, the sensor employed, etc.).

**Figure 5:**
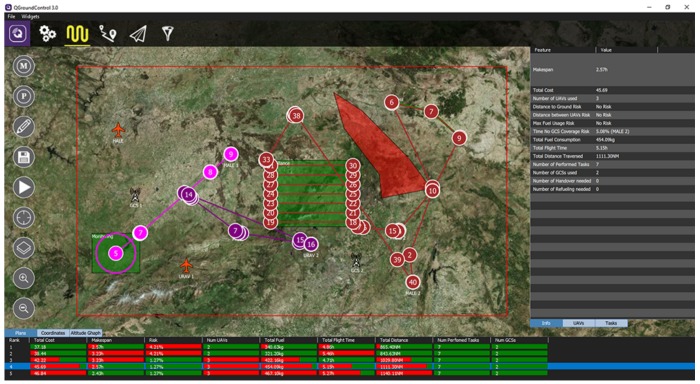
Mission plans.

After this, the new set of ranked-filtered solutions will be returned as a JSON message to QGroundControl, which decodes this message and presents the set of plans in a table at the bottom of the scenario (see Figure 5). This table shows the different objectives optimized in a percentage bar, where greener bars represent better values for the variable while redder bars represent worse values for the variable.

### 4.4. Plan Visualization

Once the plans have been computed, it is possible to view the paths and some information about a specific plan by clicking on it. The path for each UAV used in the mission is represented with a different color (see Figure 5). On the other hand, the right pop-up shows three tabs with different information about the plan. The info tab shows the different values for the objective variables and risk factors of the mission plan.

The *UAVs* tab shows, for every UAV used in the mission (represented in columns), information about the UAV assignments and performance:

- The assigned tasks (in order) that the UAV performs.
- The GCS assigned to the UAV in the mission.
- The departure time for the UAV in the mission.
- The return time for the UAV in the mission and the return flight profile (FP) used.
- The cost of use of the UAV in the mission.
- The flight time of the UAV in the mission.
- The distance traversed by the UAV in the mission.
- The fuel consumed by the UAV in the mission.
- The percentage of fuel usage of the UAV.
- The minimum distance to the ground of the UAV during the mission.
- The time spent by the UAV out of the GCS coverage during the mission.

On the other hand, if clicking on the Tasks tab, a table will appear showing the task assignments in files, where each file provides:

- The objective considered
- The task of the objective considered (at least one)
- The UAV assigned to the task.
- The departure time for the UAV when it starts going to the task zone.
- The flight profile used in the path to reach the task zone.
- The wait loiter duration in case the task has some time restriction and the vehicle must wait until its start.
- The start time of the task.
- The sensor used for the performance of the task.
- The duration of the task.
- The end time of the task.

By left clicking one of the UAVs used in the mission plan, only the path for this UAV will be represented. In addition, if there exist some out-of-coverage points for the route of the UAV, these will be represented in red. The tables of the right panel will also adapt to this selection and only show the concrete UAV and its tasks assigned.

When clicking the Coordinates tab in the bottom, a table with the different waypoints of the route of the selected UAV will show every specific parameter of each waypoint, such as the speed, time, the task associated with it, etc.

On the other hand, when clicking the Altitude Graph tab on the bottom, an altitude profile for the selected UAV will appear, including the ground altitude.

## 5. Mission Execution and Replanning

After visualizing the ranked plans, the operator selects the best one according to its criteria (should be usually the first one), and this plan will be simulated by clicking the play button on the left panel (see Figure 2).

After this, several instances of the ArduPilot program will be executed (as many as UAVs used in the mission). Each instance will be connected to the QGroundControl through a MAVLink connection, and the different UAV figures will be associated with the position of the related ArduPilots. Therefore, the UAVs will start departing according to the plan.

As the waypoints in the path for each vehicle are passed by, they will turn into a darker color, and their border will become black. The current waypoint where the UAV is going is highlighted in green. On the other hand, the tasks that finish their performance become more translucent.

During the execution of the mission, if the operator receives any external notification about an event involving a new objective, he/she can enter edit mode and add new objectives, as explained in Section 3.

Once the new tasks are added, the mission planner can be executed similarly as in pre-planning. As a previous plan is being simulated, the JSON message sent to the mission planner must contain this previous plan, so the planner knows that it must work in replanning mode. In addition, the operator introduces a concrete time for the planning process. This time will not only limit the planning process, but also will be considered as the moment where the replanning process is performed (i.e., the status of the mission passed to the replanner will be the one taking place those seconds after the actual moment).

Then, as in the planning process explained in the previous section, the DSS ranks and filters the solutions, and these are returned to the operator, where the different paths and assignments can be seen as before.

Finally, once the operator selects the new plan to be updated, the paths and assignments for the actual execution of the mission plan will be updated.

## 6. Use Cases on the Extended QGroundControl

In this section, we design two use cases to prove the new functionalities added to QGroundControl. In the first one, a general walk through of the different tools developed is done, creating a mission, planning, simulating and replanning it. In the second one, a mission that is impossible to solve is presented, in order to show how the planner informs the operator in this situation.

In order to use the test bed interface, a novel mission planning algorithm [8] has been used. This algorithm extends Non-dominated Sorting Genetic Algorithm-II (NSGA-II) [24] to focus the search on “knee point” [25], thereby looking for the most significant solutions in the POF. This approach checks the validity of solutions through a Constraint Satisfaction Problem (CSP) model developed using Gecode [26], which is connected to the fitness function of the algorithm. Moreover, the replanning algorithm used [7] is the same approach as mission planning, but taking into account the previous plan and the limited time for the algorithm.

On the other hand, for the DSS, VIKOR [27] has been used to rank the solutions returned by the MOEA, using the factors defined by the operator profile as the weights of the criteria. The VIKOR method uses the Manhattan distance and the Chebyshev distance and provides a compromise solution, considering the maximum utility and the minimum individual regret. Finally, the filtering is performed through a distance function that assigns a weight to each variable based on its importance. When two solutions are separated less than a filter threshold, the one with the lower rank value is omitted. The resultant set of ranked and filtered solutions is then returned through the Thrift interface.

### 6.1. First Use Case: A Walk through the Framework

**Figure A1:**
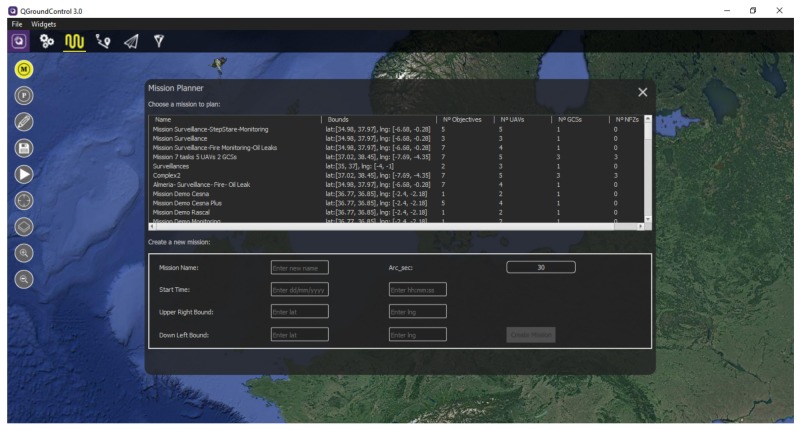
Panel in the QGroundControl for reading or creating missions.

First, to create a new mission, we use the “M” button in the left panel to access the read or create mission panel (see Figure A1). Here, we must provide the parameters mentioned in Section 3.1. In this case, the bounds are latitude between 36.76^°^ and 36.85^°^ and longitude between −2.396^°^ and −2.174^°^, and 7.5 arc-seconds are used in the elevation map. The start time of the mission is not specified. Then, by clicking the Create Mission button (it will be available as long as all the mandatory values are fulfilled and correct), the mission will be created, and an empty scenario inside the defined bounds will be presented.

**Figure A2:**
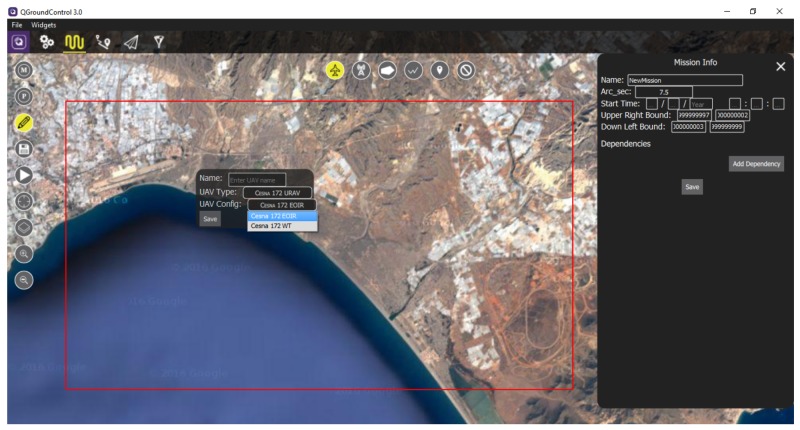
Adding a new UAV in the mission designer.

Now, to recreate the mission presented in Figure 2, it is necessary to add the objectives, UAVs, GCSs and NFZs. These instances are added using the Mission Designer, selecting the Edit button represented with a pencil in the left panel. Then, the set of icons at the top of the scenario is used to add each of the elements of the mission. In this case, we consider 4 UAVs, 1 GCS, 1 NFZ and 5 objectives (monitoring, surveillance, patrol, tracking and target photographing). To add the vehicles, just clicking on the corresponding icon at the top and then on the desired position in the scenario, the elements will be positioned, and a pop-up will appear to specify the mandatory properties of these elements (e.g., Figure A2 shows this pop-up for a UAV, where a name for this vehicle must be provided, as well as its type and configuration). For the GCSs and the objectives, this pop-up will require a name and the type of the station or the objective, while NFZs do not require any parameter, so no pop-up will appear when creating them. As was mentioned in Section 3.4, when adding zone and path objectives and also when adding NFZs, after clicking the corresponding icon for these elements, a set of points in the scenario must be clicked in order to create the desired zone or path.

If the elements created are not properly positioned, all of them can be dragged and, thus, their positions and vertices updated. In addition, each vertex for path and zone objectives can be moved using the orange points that appear at each vertex in edit mode. The entry point (yellow) and exit point (red) for zone objectives can also be dragged. On the other hand, any element of the scenario can be deleted by just right clicking over it.

**Figure A3:**
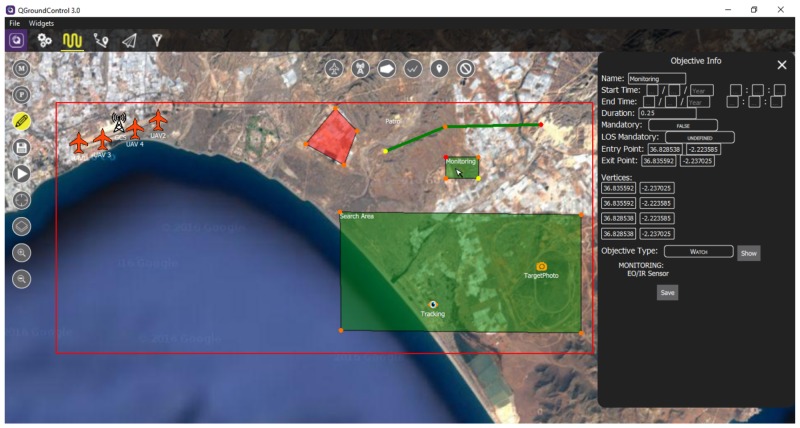
Modifying zone objective properties in the mission designer.

Once the elements have been created, to modify their different properties, just left clicking on them will trigger a panel in the right, showing the properties of the element (e.g., Figure A3 shows the properties of the monitoring objective, where entry and exit points have been added and a duration of 15 min has been established). Once these properties are modified, the Save button must be clicked and a message “Saved successfully” must appear or an error message indicating the possible error. The tracking objective has a duration of 10 min, and the rest of the objectives have not been modified. On the other hand, the UAVs considered are a HALE (with Electro-optical or Infra-red (EO/IR) camera and Synthetic Aperture Radar (SAR) radar) two URAV (with EO/IR camera) and a MAlE(with Maritime Patrol Radar (MPR) and SAR radars).

When first entering edit mode, a general Mission Info panel appears on the right, where the initial definition of the mission (bounds, start time, etc.) can be modified. In this panel, dependencies between objectives can also be added by clicking the Add Dependency button and deleted with the “X” button above the concrete dependency. As can be seen in Figure 2, we consider two dependencies for this mission, establishing that the surveillance objective (search area) must precede the target photographing and tracking objectives, and the tracking objective must be performed by the same UAV that performs the surveillance. To save the new dependencies added or deleted, the Save button of the panel must be clicked.

Once the entire mission has been defined, in order to save it in the database, the Save button on the left (the one with the floppy disk) is used.

**Figure A4:**
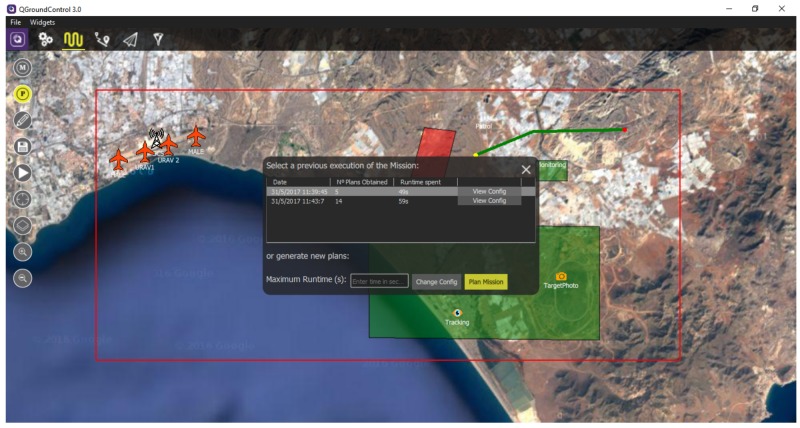
Using the automated mission planner.

Once the mission is defined, the mission planner can be executed in order to find solutions for this mission. This is done by clicking the “P” button on the left (see Figure A4). Then, a pop-up will appear showing previous executions of the mission planner at the top and a button for executing the planner (including a box for indicating the maximum runtime) at the bottom.

**Figure A5:**
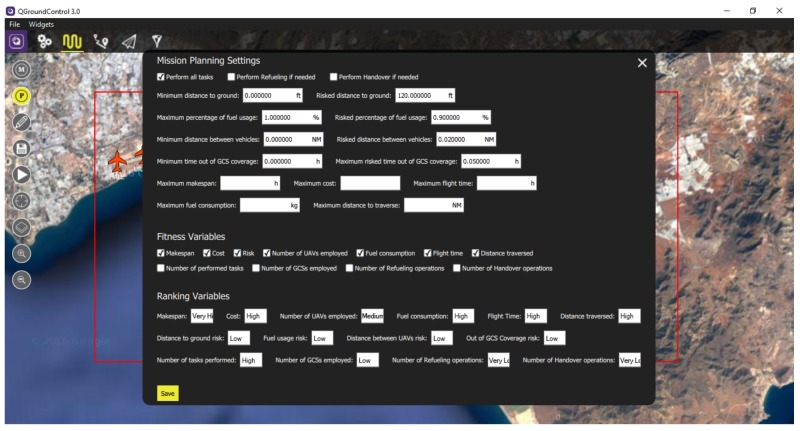
Changing the operator settings for the mission.

Additionally, the Change Configbutton permits one to change the operator preferences defined in Section 4.1 for this concrete mission (see Figure A5). In this case, the ranking values are assigned a very high value for the makespan; high values for cost, fuel consumption, flight time, distance and number of tasks performed; a medium value for the number of vehicles used; and a low value for the risk factors and the number of stations used. When clicking the Plan Mission button, the automated mission planner starts running with the mission designed.

**Figure A6:**
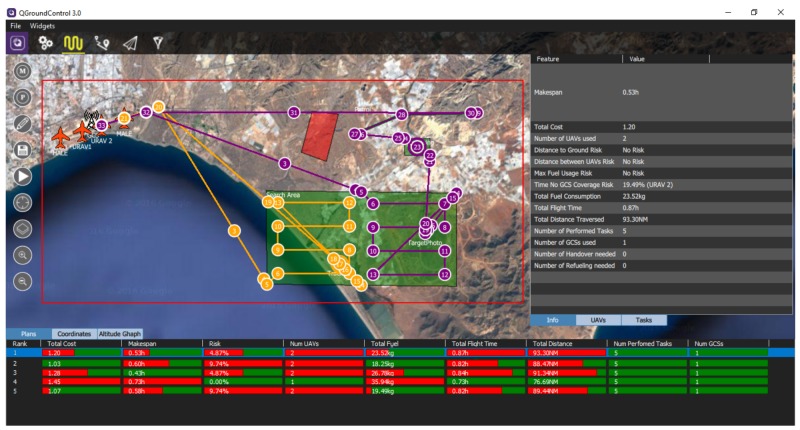
Mission plan routes and info.

After the mission planner gets the solutions, and the DSS ranks and filters them, they are presented in a table at the bottom of the scenario (see Figure A6). Each row of the table represent a solution, and each column represents the value of an optimization variable. The cells are filled as a percentage bar, where the greener a cell is, the better the optimization variable for that solution with respect to the others. When clicking one of the rows of this table, the paths for each vehicle will be shown, and a panel on the right will appear presenting some information about the plan, including the risk factors. This panel has two extra tabs: the UAVs tab presents information about the performance of every specific UAV, including the departure time of the vehicle, the fuel consumed, the assigned tasks, etc.; while the Tasks tab presents information about every task, including the vehicles performing it, the flight profile used by them, the sensor employed, etc.

**Figure A7:**
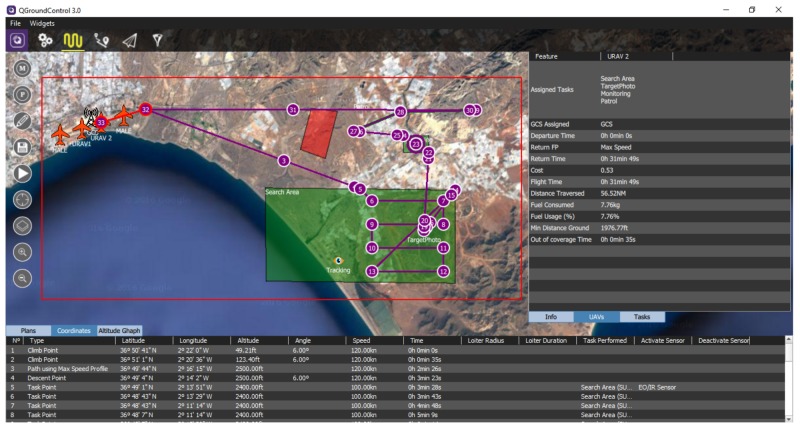
Route for URAV 2, including the waypoint table and UAV info.

When selecting one of the UAVs used in the plan, only the path for this UAV will be represented (see Figure A7). In this situation, the Coordinates tab in the bottom will present a table with the different waypoints of the selected vehicle, including parameters such as the speed, estimated time of arrival, etc.

**Figure A8:**
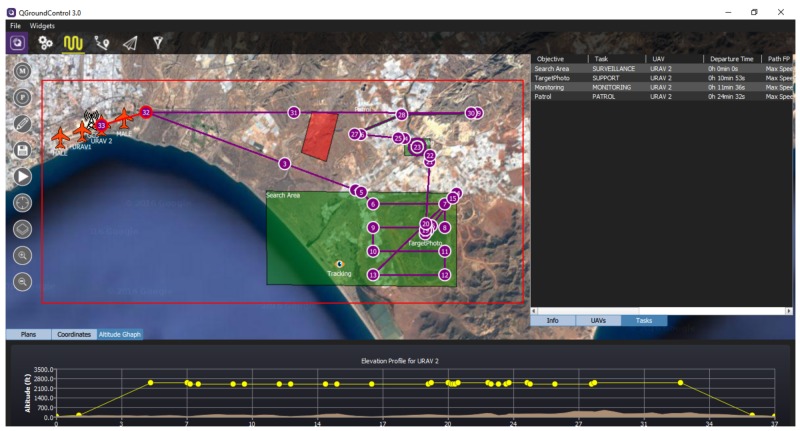
Altitude Profile for URAV 2 and the task table.

On the other hand, when clicking the Altitude Graph tab on the bottom (see Figure A8), an altitude profile for the selected UAV (in yellow) will appear, including the ground altitude (in brown).

**Figure A9:**
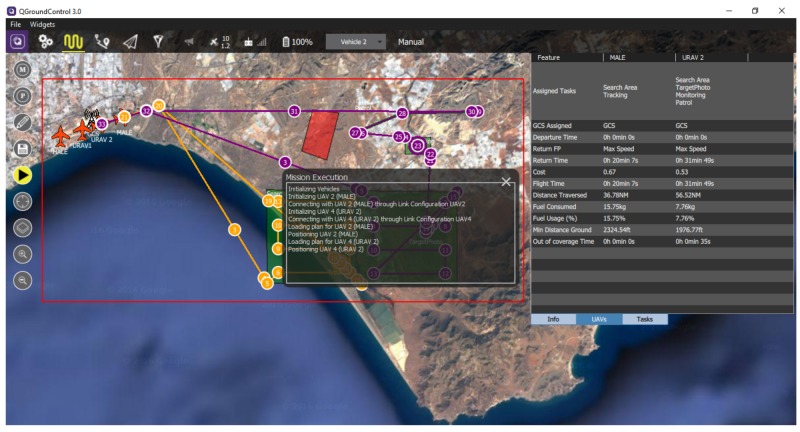
Mission execution.

Now that the returned plans have been studied, we select the best one, which usually should be the first one as in this case. Then, to simulate this plan, we click the play button on the left panel (see Figure A9). Then, a pop-up indicates that the ArduPilots simulating the vehicles are being initialized and the paths are being loaded to them.

**Figure A10:**
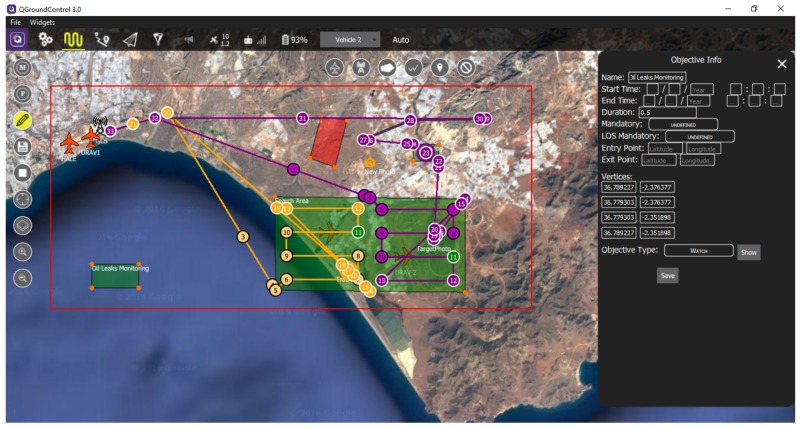
Mission online edition. Adding new objectives during execution.

Once the simulation starts, it can be seen how the vehicles in the scenario start moving and the waypoints where they are going are marked in green. Once these waypoints are traversed, their color becomes darker and their border turns black. Meanwhile, when tasks are completed, they become more translucent (see Figure A10). When the exact moment comes, in our case it is represented in Figure A10, we simulate that two new tasks must be performed as soon as possible, so we add them to our current mission. To do that, we select the Edit button and add new objectives. In this case, oil leaks monitoring and new photo objectives have been added.

**Figure A11:**
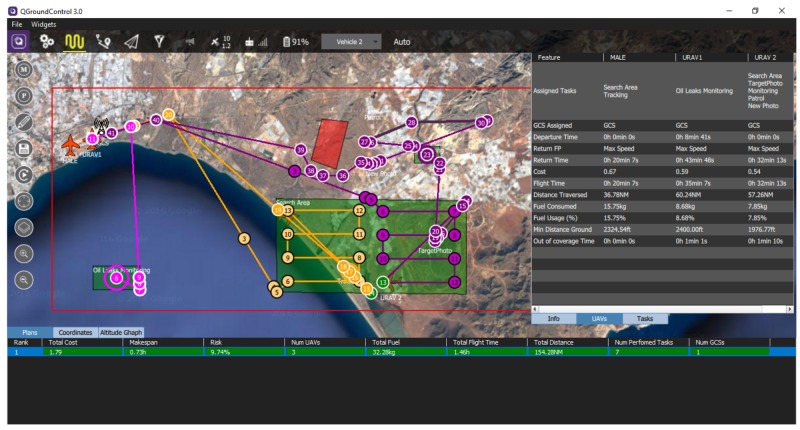
Solution of the mission replanning.

After the online editing, we click the Planner button and introduce a concrete time for the mission planning process (usually 1 or 2 min). Then, by clicking the Plan Mission button, the mission replanner will be executed during the specified time. When this process finishes, new solutions will be returned in the form of a table (see Figure A11), where the different paths and assignments can be seen as before.

**Figure A12:**
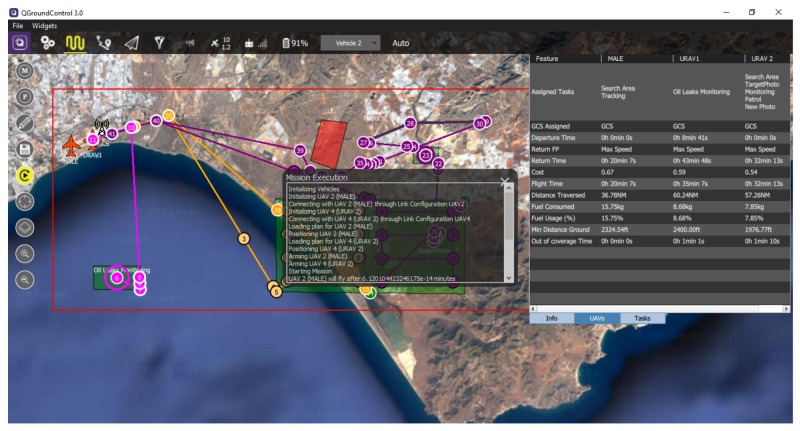
Mission update with the replanned solution.

In this case, the optimization process has returned one solution. This new plan involves a new vehicle to perform the oil leaks monitoring objective and the re-routing of URAV 2 for performing the new photo objective. Now, we click the Re-Execute button (see Figure A12), and the paths and assignments for the actual executing mission plan will be updated.

### 6.2. Second Use Case: Working with Unresolvable Missions

**Figure A13:**
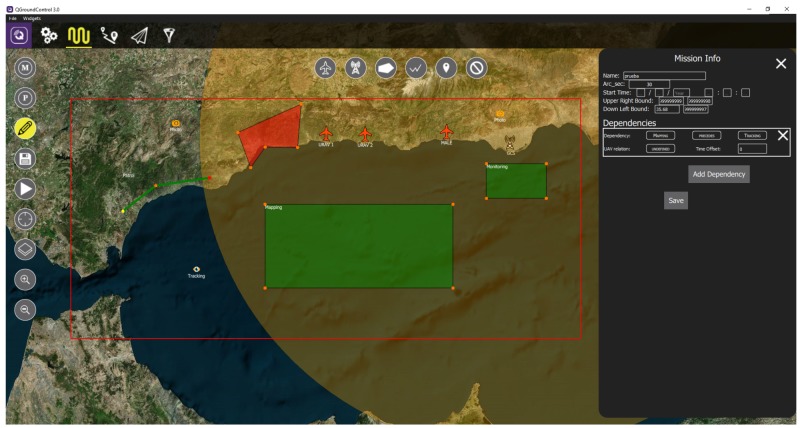
Mission considered in the second use case.

In this case, we consider a new mission, represented in Figure A13, with 3 UAVs, 1 GCSs, 1 NFZ and 6 objectives. As can be seen, the mission cannot be performed because the range of the GCS does not cover all the objectives of the mission.

**Figure A14:**
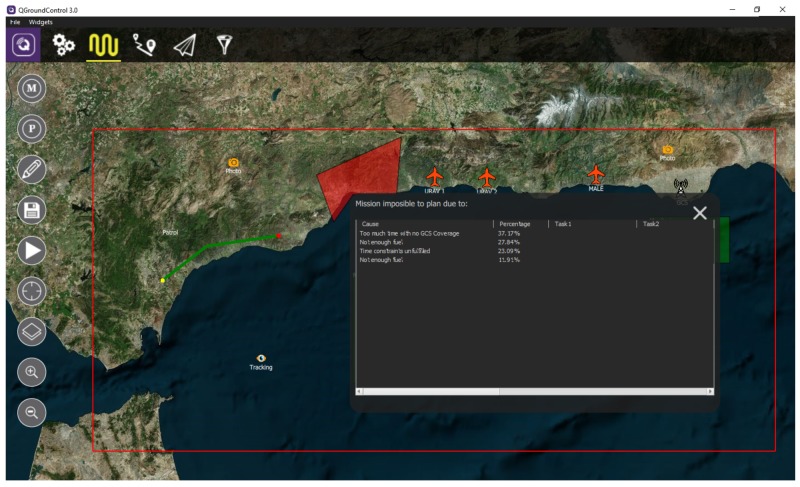
No solutions obtained for the mission in the second use case.

In this case, when performing the planning process, following the same steps as in the previous case, the mission planner will not return any solution. Instead, a pop-up will appear (see Figure A14), informing that no solution was found, as well as the errors that occurred most frequently during the checking in the CSP model, so they represent the most probable problem that presents this mission, so the operator can reformulate the mission to make it feasible. As can be seen in Figure A14, the planner informs that the main problem is that the vehicles spent too much time out of the coverage of the GCS, as was pointed out before. Furthermore, it is appreciable that the fuel of the vehicles may be insufficient in some plans.

## 7. Conclusions

In this work, we have extended the QGroundControl framework, adding the functionalities defined in Figure 1. The contribution of this work consists of:

1. The design and development of a mission designer, which provides an interactive environment for the creation and visualization of missions, including its objectives/tasks, vehicles, GCSs, NFZs, etc.
2. Integration of an interface for an automated mission planner and DSS, in order to test different mission planning and DSS algorithms, which generate, rank and filter plans for the missions designed.
3. Design and development of a plan visualizer, which permits one to graphically represent the plans, including the paths for each UAV and the information related to the optimality and risks of the plan.
4. Design and development of a mission monitoring system, which informs the operator about the waypoints already passed by and the tasks already performed.
5. Design and development of a replanning system and integration of the automated mission replanner inside the QGroundControl by reusing the interface for automated mission planning. This permits the operator to inform the system about new objectives/tasks or incidents during the execution of the mission and call the mission replanner in order to obtain new plans for the updated mission.

In future works, this framework will be outperformed adding other novel techniques that are being developed for UAVs, such as a training system for operators, the use of new controlling devices (e.g., virtual reality glasses or motion sensing devices) or the inclusion of augmented reality in the simulation.

## Figures and Tables

**Table 1:** Allen’s interval algebra.

Relation	Illustration	Interpretation
$T_{1}<T_{2}$	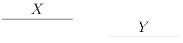	$T_{1}$ takes place before $T_{2}$
$T_{1}\;m\;T_{2}$	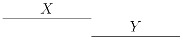	$T_{1}$ meets $T_{2}$
$T_{1}\;o\;T_{2}$	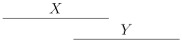	$T_{1}$ overlaps $T_{2}$
$T_{1}\;s\;T_{2}$	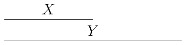	$T_{1}$ starts $T_{2}$
$T_{1}\;d\;T_{2}$	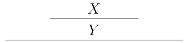	$T_{1}$ during $T_{2}$
$T_{1}\;f\;T_{2}$	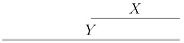	$T_{1}$ finishes $T_{2}$
$T_{1}=T_{2}$	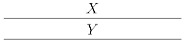	$T_{1}$ is equal to $T_{2}$

## Appendix A. Use Cases (Screenshots)

Here, we provide the different screenshots from the simulator execution, used in the use cases presented in Section 6.
